# Supplementary material for: Mood Disorders and Gluten: It’s Not All in Your Mind! A Systematic Review with Meta-Analysis
Source: Nutrients. 2018 Nov 8;10(11):1708. doi: 10.3390/nu10111708 (PMC6266949; doi:10.3390/nu10111708)
Supplement: Supplementary file 1 [file nutrients-10-01708-s001.zip › nutrients-380101-supplementary proof/Supplementary File 1 Checked.docx]

Supplementary Material 1: Search strategies

Table S1. Medline (via EBSCOhost)

|  | Search terms | Results |
| --- | --- | --- |
| 1 | (MP "Diet, Gluten-Free") OR “gluten-free diet” |  |
| 2 | (MP ((gluten OR wheat) N2 sensitivity) |  |
| 3 | (TI “wheat-free”) or (AB “wheat-free”) |  |
| 4 | (TI gluten N3 challenge) OR (AB gluten N3 challenge) |  |
| 5 | (TI wheat N3 challenge) OR (AB wheat N3 challenge) |  |
| 6 | 1 OR 2 OR 3 OR 4 OR 5 |  |
| 7 | (MH Depression) OR (TX depress*) |  |
| 8 | (MP "Affective Symptoms") |  |
| 9 | (MH "Aggression”) |  |
| 10 | (MP "Irritable Mood") |  |
| 11 | (MH "Mood Disorders+") |  |
| 12 | (MP "Seasonal Affective Disorder") |  |
| 13 | (MP "Cyclothymic Disorder") |  |
| 14 | (MP "Depressive Disorder+") |  |
| 15 | (MH "Bipolar and Related Disorders+") |  |
| 16 | (MP "Anger+") |  |
| 17 | (MP “psychological general well-being”) |  |
| 18 | (MP PHQ-9) |  |
| 19 | (MP (“profile of mood states”) |  |
| 20 | 7 OR 8 OR 9 OR 10 OR 11 OR 12 OR 13 OR 14 OR 15 OR 16 OR 17 OR 18 OR 19 |  |
| 21 | 6 AND 20 | 155 |
| 22 | 21 AND ((MP (prospective OR longitudinal OR trial OR randomi?ed OR placebo OR "healthy controls" OR "one year" OR “follow-up”)) NOT (MP “cross-sectional”)) | 48 |

Table S2. CINAHL (via EBSCOhost)

|  | Search terms | Results |
| --- | --- | --- |
| 1 | "Diet, Gluten-Free” OR "gluten free diet" OR "Gluten Adverse Effects" |  |
| 2 | ((gluten OR wheat) N2 sensitivity) |  |
| 3 | “wheat-free” |  |
| 4 | ((gluten OR wheat) N3 challenge) |  |
| 5 | 1 OR 2 OR 3 OR 4 |  |
| 6 | (MH "Affective Disorders+") |  |
| 7 | (MH "Depression+") OR (TX depress*) |  |
| 8 | (MH "Bipolar Disorder+") OR bipolar |  |
| 9 | (MH "Dysthymic Disorder") OR dysthmi* |  |
| 10 | (MH "Cyclothymic Disorder") OR cyclothymi* |  |
| 11 | (MH "Seasonal Affective Disorder") |  |
| 12 | (MH "Affective Symptoms+") |  |
| 13 | (MH aggressi*) |  |
| 14 | (MH "Affect") OR mood |  |
| 15 | (Anger OR angry) |  |
| 16 | (MH "Hamilton Rating Scale for Depression") |  |
| 17 | (Depression N2 Scale) |  |
| 18 | (MH "Beck Depression Inventory, Revised Edition") OR “Beck Depression Inventory” |  |
| 19 | "Profile of Mood States" |  |
| 20 | “Psychological General Well-Being” |  |
| 21 | “PHQ-9” |  |
| 22 | 6 OR 7 OR 8 OR 9 OR 10 OR 11 OR 12 OR 13 OR 14 OR 15 OR 16 OR 17 OR 18 OR 19 OR 20 OR 21 |  |
| 23 | 5 AND 22 | 154 |
| 24 | 23 AND ((prospective OR longitudinal OR trial OR randomi?ed OR OR placebo OR "healthy controls" OR "follow up" OR "one year" OR “1 year”) NOT ((AB "cross-sectional") OR (TI "cross-sectional"))) | 18 |

Table S3. Web of Science (via EBSCOhost)

|  | Search terms | Results |
| --- | --- | --- |
| 1 | "Diet, Gluten-Free” OR "gluten free diet" OR "Gluten Adverse Effects" |  |
| 2 | ((gluten OR wheat) N2 sensitivity) |  |
| 3 | “wheat-free” |  |
| 4 | ((gluten OR wheat) N3 challenge) |  |
| 5 | 1 OR 2 OR 3 OR 4 |  |
| 6 | (MH "Affective Disorders+") |  |
| 7 | (MH "Depression+") OR (TX depress*) |  |
| 8 | (MH "Bipolar Disorder+") OR bipolar |  |
| 9 | (MH "Dysthymic Disorder") OR dysthmi* |  |
| 10 | (MH "Cyclothymic Disorder") OR cyclothymi* |  |
| 11 | (MH "Seasonal Affective Disorder") |  |
| 12 | (MH "Affective Symptoms+") |  |
| 13 | (MH aggressi*) |  |
| 14 | (MH "Affect") OR mood |  |
| 15 | (Anger OR angry) |  |
| 16 | (MH "Hamilton Rating Scale for Depression") |  |
| 17 | (Depression N2 Scale) |  |
| 18 | (MH "Beck Depression Inventory, Revised Edition") OR “Beck Depression Inventory” |  |
| 19 | "Profile of Mood States" |  |
| 20 | “Psychological General Well-Being” |  |
| 21 | “PHQ-9” |  |
| 22 | 6 OR 7 OR 8 OR 9 OR 10 OR 11 OR 12 OR 13 OR 14 OR 15 OR 16 OR 17 OR 18 OR 19 OR 20 OR 21 |  |
| 23 | 5 AND 22 | 245 |
| 24 | 23 AND ((prospective OR longitudinal OR trial OR randomi?ed OR OR placebo OR "healthy controls" OR "follow up" OR "one year" OR “1 year”) NOT ((AB "cross-sectional") OR (TI "cross-sectional"))) | 56 |
| 25 | Limit 25 – Scholarly (Peer Reviewed) Journals; Document Type: Article | 54 |

Table S4. PsycINFO (via EBSCOhost)

|  | Search terms | Results |
| --- | --- | --- |
| 1 | “gluten-free diet” |  |
| 2 | “wheat-free” |  |
| 3 | (TI gluten N5 challenge) OR (AB gluten N5 challenge) |  |
| 4 | (TI wheat N5 challenge) OR (AB wheat N5 challenge) |  |
| 5 | 1 OR 2 OR 3 OR 4 |  |
| 6 | "mood disorder*" |  |
| 7 | depress* |  |
| 8 | (DE "Affective Disorders”) |  |
| 9 | (DE "Bipolar Disorder") |  |
| 10 | (DE "Dysthymic Disorder") |  |
| 11 | (DE "Cyclothymic Disorder") |  |
| 12 | (DE "Seasonal Affective Disorder") |  |
| 13 | "affective symptom*" |  |
| 14 | (DE "Major Depression") |  |
| 15 | (DE "Irritability") |  |
| 16 | (DE "Depression (Emotion)") |  |
| 17 | psychological N2 “well-being" |  |
| 18 | (TM depress*) |  |
| 19 | "Profile of Mood States" |  |
| 20 | “PHQ-9” |  |
| 21 | 6 OR 7 OR 8 OR 9 OR 10 OR 11 OR 12 OR 13 OR 14 OR 15 OR 16 OR 17 OR 18 OR 19 OR 20 |  |
| 22 | 5 AND 21 |  |
| 23 | Limit 22 to - Publication Type: All Journals |  |
| 24 | 23 AND ((prospective OR longitudinal OR trial OR randomi?ed OR OR placebo OR "healthy controls" OR "follow up" OR "one year" OR “1 year” OR “months”) NOT ((AB "cross-sectional") OR (TI "cross-sectional"))) | 7 |

Table S5. Scopus

|  | Search terms | Results |
| --- | --- | --- |
| 1 | TITLE-ABS-KEY("Diet, Gluten-Free") |  |
| 2 | TITLE-ABS-KEY(“gluten-free diet”) |  |
| 3 | TITLE-ABS-KEY(gluten W/2 sensitivity) |  |
| 4 | TITLE-ABS-KEY(“wheat-free”) |  |
| 5 | TITLE-ABS-KEY(wheat W/2 sensitivity) |  |
| 6 | TITLE-ABS-KEY(gluten W/3 challenge) |  |
| 7 | TITLE-ABS-KEY(wheat W/3 challenge) |  |
| 8 | 1 OR 2 OR 3 OR 4 OR 5 OR 6 OR 7 |  |
| 9 | TITLE-ABS-KEY(“mood disorder*”) |  |
| 10 | TITLE-ABS-KEY(“affective disorder*” OR “affective symptom*”) |  |
| 11 | TITLE-ABS-KEY(depress*) |  |
| 12 | TITLE-ABS-KEY(“bipolar disorder”) |  |
| 13 | TITLE-ABS-KEY(dysthymi*) |  |
| 14 | TITLE-ABS-KEY(cyclothymi*) |  |
| 15 | TITLE-ABS-KEY(“seasonal affective disorder") |  |
| 16 | TITLE-ABS-KEY(aggressi*) |  |
| 17 | INDEXTERMS("Irritable Mood" OR Irritability) |  |
| 18 | TITLE-ABS-KEY(anger OR angry) |  |
| 19 | TITLE-ABS-KEY(“psychological general well-being”) |  |
| 20 | TITLE-ABS-KEY(“PHQ-9”) |  |
| 21 | TITLE-ABS-KEY(“profile of mood states”) |  |
| 22 | 9 OR 10 OR 11 OR 12 OR 13 OR 14 OR 15 OR 16 OR 17 OR 18 OR 19 OR 20 OR 21 |  |
| 23 | 6 AND 29 | 351 |
| 24 | 23 AND (LIMIT-TO(EXACTKEYWORD, (*"Human" OR “Humans”)*) | 316 |
| 25 | 24 AND (LIMIT-TO(DOCTYPE, *"ar"*) | 219 |
| 26 | 25 AND ((TITLE-ABS-KEY(prospective OR longitudinal OR trial OR randomi?ed OR placebo OR "healthy controls" OR "follow up" OR "one year")) AND NOT (TITLE-ABS-KEY(“cross-sectional))) | 84 |

Table S6. Cochrane Library

|  | Search terms | Results |
| --- | --- | --- |
| 1 | ([mh "Diet, Gluten-Free"] OR “gluten-free diet”):ti,ab,kw |  |
| 2 | ((gluten OR wheat) NEAR/2 sensitivity):ti,ab,kw |  |
| 3 | (“wheat-free”):ti,ab,kw |  |
| 4 | (gluten NEAR/3 challenge):ti,ab,kw |  |
| 5 | (wheat NEAR/3 challenge):ti,ab,kw |  |
| 6 | 1 OR 2 OR 3 OR 4 OR 5 |  |
| 7 | [mh Depression] OR depress* |  |
| 8 | "Affective Symptoms" |  |
| 9 | [mh Aggression] |  |
| 10 | "Irritable Mood" |  |
| 11 | [mh "Mood Disorders"] |  |
| 12 | "Seasonal Affective Disorder" |  |
| 13 | "Cyclothymic Disorder" |  |
| 14 | "Depressive Disorder" |  |
| 15 | [mh "Bipolar and Related Disorders"] |  |
| 16 | Anger |  |
| 17 | “psychological general well-being” |  |
| 18 | “PHQ-9” |  |
| 19 | “profile of mood states” |  |
| 20 | 7 OR 8 OR 9 OR 10 OR 11 OR 12 OR 13 OR 14 OR 15 OR 16 OR 17 OR 18 OR 19 |  |
| 21 | 6 AND 20 |  |
| 22 | 21 AND ((prospective OR longitudinal OR trial OR randomi*ed OR placebo OR "healthy controls" OR "one year" OR “follow-up”) NOT (“cross-sectional”)):ti,ab,kw | 25 |
