# Supplementary material for: Mood Disorders and Gluten: It’s Not All in Your Mind! A Systematic Review with Meta-Analysis
Source: Nutrients. 2018 Nov 8;10(11):1708. doi: 10.3390/nu10111708 (PMC6266949; doi:10.3390/nu10111708)
Supplement: Supplementary file 1 [file nutrients-10-01708-s001.zip › nutrients-380101-supplementary proof/Supplementary File 2 Edited.docx]

Table S7. Specific criteria for assessing the risk of bias (RoB) for each of the domains from Cochrane’s RoB 2.0 and ROBINS-I tools in the context of our review

| **RoB Domain** | **Specific Criteria** |
| --- | --- |
| Bias due to confounding | In terms of bias due to confounding, factors specific to the outcomes of interest were participant exposure to a gluten-free diet (GFD) prior to enrolment into the study, participant use of antidepressant medication, and modification of questionnaires to avoid bias due to GI symptoms, eating habits and/or appetite. Hence, studies that did not report the diet of subjects prior to participation in their study and/or participant use of antidepressants before/during the study, and used unmodified questionnaires to assess mood containing questions related to GI symptoms and/or appetite, were therefore at an increased RoB in this context. |
| Selection bias | A further confounding factor is compliance to the GFD, as the primary aim of this SR is to assess the effect of adhering to a GFD on the outcome of depressive symptoms. Hence, in terms of selection bias, although the ROBINS-I tool suggests that participants excluded due to characteristics observed after the start of intervention increases the RoB, studies excluding participants based on non-adherence to the GFD did not increase the RoB in the context of this review. On the other hand, studies could include non-adherent participants, but report results of depressive outcomes separate to adherent participants in order to avoid bias due to confounding. |
| Bias in the classification of interventions | Studies were deemed at an increased RoB in terms of the classification of intervention groups if the methods for classifying participants were based on retrospective data or subjective measures. For example, studies classifying participants as having coeliac disease (CD) or not must adequately describe objective methods based on serological and histological measures using Marsh classifications to remain at a low RoB due to classification. |
| Bias due to deviations from intended interventions | In terms of measuring adherence, studies relying solely on unvalidated subjective methods, histology or inadequately described objective methods were at an increased RoB. Firstly, it is known that subjective reporting can introduce significant bias, especially if participants are wanting to ‘please’ the physicians or study researchers. Secondly, is has been suggested that serology is an unreliable measure to assess adherence to a GFD in those with CD*. Moreover, RoB also increased for studies that did not quantitatively report the level of adherence to the GFD, studies reporting that <90% of included participants did not strictly adhere to the intervention (GFD and/or gluten challenge) or, for studies where >10% of included participants were less than strictly adherent, they did not separately analyse adherent and partially-/non-adherent participants. |
| Bias due to incomplete outcome data | Despite the Cochrane guidelines associating per-protocol (PP) analyses with an increased RoB, while intention-to-treat (ITT) analyses are normally associated with a low RoB, a PP analysis did not increase the RoB in the context of this study while an ITT analysis did. This is because, unlike a normal Cochrane systematic review, this review was not interested in all the health outcomes for the intervention. Rather, only mood symptoms based on adherence to a GFD were of interest, so studies including non-adherent participants not completing the study protocol in the overall results were at an increased RoB. Furthermore, studies that did not clearly state if the analysis was PP or ITT, or give the final numbers of participants included in the FU analysis, were treated as ITT with an increased RoB. |
| Detection bias | The outcome of interest, depressive symptoms, may only be measured subjectively. However, studies that used clinician-/researcher-administered structured interviews to assess symptoms were classified as having a low RoB, while studies that used patient-administered self-reported questionnaires were at either a moderate or serious RoB. Moreover, studies that administered self-reported questionnaires during outpatient visits and/or in a controlled/laboratory environment were seen as having a moderate RoB, while those where participants completed questionnaires at home were at serious RoB. If the method of administration was not adequately described, the RoB was classed as serious for this dimension of the study. |
| Reporting bias | No modifications or special criteria were applied to this aspect of the RoB tool. Studies were at an increased RoB if clearly measured data were not published in the results. |
