# Supplementary material for: Mood Disorders and Gluten: It’s Not All in Your Mind! A Systematic Review with Meta-Analysis
Source: Nutrients. 2018 Nov 8;10(11):1708. doi: 10.3390/nu10111708 (PMC6266949; doi:10.3390/nu10111708)
Supplement: Supplementary file 1 [file nutrients-10-01708-s001.zip › nutrients-380101-supplementary proof/Supplementary File 3 Edited.docx]

Table S8. Studies excluded by full-text screening

| Author | Year | Reason for exclusion* |
| --- | --- | --- |
| Addolorato *et al.* | 2004 | 2 - no baseline scores |
| Black and Orfila | 2011 | 3 - cross-sectional survey |
| Brottveit *et al.* | 2012 | 2 - only HADS score at baseline/no FU |
| Burger *et al.* | 2017 | 1 - only total QoL and SF-36 |
| Caio *et al.* | 2014 | 2 - did not publish extra-intestinal symptom scores |
| Chauhan *et al.* | 2010 | 3 - retrospective |
| Ciacci *et al.* | 2007 | 7 |
| Ciacci *et al.* | 2002 | 3 - retrospective |
| Dieterich *et al.* | 2018 | 7 |
| Dipper *et al.* | 2009 | 1 |
| Elli *et al.* | 2016 | 1 - only SF-36 MCS |
| Fuchs *et al.* | 2018 | 6 - irrelevant post-hoc subgroup analysis of included study (Ukkola *et al*. 2011) |
| Jacobsson *et al.* | 2012 | 5 |
| Kaukinen *et al.* | 2007 | 2 |
| Kelly *et al.* | 2013 | 2 |
| Kurppa *et al.* | 2008 | 3 - case report |
| Levran *et al.* | 2018 | 5 - 50% children report eating foods containing gluten residues |
| Lichtwark *et al.* | 2014 | 2 - STPI scores not reported |
| Mansikka *et al.* | 2018 | 3 - retrospective |
| Mustalahti *et al.* | 2002 | 2 - did not publish separate PGWB subcategory scores |
| Nordyke *et al.* | 2013 | 1 - EQ-5D questionnaire did not differentiate between anxiety/depression |
| Paavola *et al.* | 2012 | 3 - retrospective |
| Pasternack *et al.* | 2017 | 6 - irrelevant post-hoc subgroup analysis of included study (Ukkola *et al*. 2011) |
| Pennisi *et al.* | 2017 | 3 - retrospective |
| Peters *et al.* | 2016 | 8 - only conference abstract |
| Peräaho *et al.* | 2003 | 2 - did not publish separate PGWB subcategory scores |
| Pouchot *et al.* | 2014 | 2 - no baseline scores |
| Pynnönen *et al.* | 2005 | 4 |
| Rajani *et al.* | 2016 | 1 |
| Rodrigo *et al.* | 2014 | 1 - only SF-36 MCS |
| Russo *et al.* | 2017 | 1 - only measures dysphoria |
| Sainsbury, Mullan and Sharpe | 2015 | 1 |
| Shalimar *et al.* | 2012 | 1 |
| Slim *et al.* | 2017 | 4 |
| Suakkonen *et al.* | 2016 | 6 - irrelevant post-hoc subgroup analysis of included study (Ukkola *et al*. 2011) |
| Turco *et al.* | 2011 | 2 - only CDI at baseline |
| Ukkola *et al.* | 2012 | 1 |
| Volta *et al.* | 2014 | 3 - cross-sectional survey |

* 1 – wrong outcome/depression not measured; 2 – depression measured but necessary scores/sub-scores not reported; 3 – wrong study design (cross-sectional/case report/retrospective); 4 – dietary adherence not measured; 5 – dietary adherence <70% and no subgroup analysis; 6 – not original study; 7 – co-interventions assessed other diets and/or medications in addition to gluten/GFD (gluten-free diet); 8 – only abstract/no published full-text.

HADS, Hospital Anxiety and Depression Scale; FU, follow-up; QoL, quality of life; SF-36, Short Form health-related quality of life questionnaire; MCS, Mental Component Score; STPI, State-Trait Personality Inventory; PGWB, Psychological General Well-Being questionnaire; EQ-5D, EuroQOL five dimensions questionnaire; CDI, Children’s Depression Inventory.
