# Supplementary material for: Mood Disorders and Gluten: It’s Not All in Your Mind! A Systematic Review with Meta-Analysis
Source: Nutrients. 2018 Nov 8;10(11):1708. doi: 10.3390/nu10111708 (PMC6266949; doi:10.3390/nu10111708)
Supplement: Supplementary file 1 [file nutrients-10-01708-s001.zip › nutrients-380101-supplementary proof/Supplementary File 5 (new).docx]

**Table S9.** Summary of findings for the long-term effects of a gluten-free diet (GFD) on depressive symptoms.

| **A GFD Compared to A Normal Gluten-Containing Diet for Depressive Symptoms (Long-Term Effects)** | | | | | | |
| --- | --- | --- | --- | --- | --- | --- |
| **Patient or population**: Any  **Intervention**: A GFD  **Comparison**: A normal gluten-containing diet  **Follow up:** Long-term (six weeks to one year)  **Study type:** Any | | | | | | |
| **Outcomes** | **Anticipated Absolute Effects * (95% CI)** | | **Relative Effect (95% CI)** | **Number of Participants  (Studies)** | **Certainty of the Evidence (GRADE)** | **Comments** |
|  | **Risk with A Normal Gluten-Containing Diet** | **Risk with A GFD** |  |  |  |  |
| Change in mean depression scores from baseline follow up: Range six weeks to one year | - | SMD 0.38 lower  (0.57 lower to 0.19 lower)  *p =* 0.0001 | - | 953  (1 RCT and 7 non-randomised studies) | ⨁◯◯◯  VERY LOW ^a,b,c,d,e^ | A GFD may reduce mean depression scores from baseline but we are very uncertain. Subgroup analyses: Differences between subgroups for CD diagnosis (CD and non-CD participants) and HLA-DQ8/2 genotype (positive or negative) were nonsignificant. |
| Difference in mean depression scores between intervention groups follow up: Mean one year | - | SMD 0.23 lower  (0.85 lower to 0.39 higher)  *p =* 0.47 | - | 40  (1 RCT) | ⨁⨁◯◯  LOW ^f,g^ | The evidence suggests that a GFD results in a small but insignificant trend towards a reduction in mean depression scores compared to a gluten-containing diet. |
| Subgroup-classical symptoms | - | SMD 0.65 lower  (0.96 lower to 0.34 lower)  *p* < 0.0001 | - | 552  (2 non-randomised studies) | ⨁◯◯◯  VERY LOW ^d,h,I,j^ | Upon subgroup analysis, the evidence suggests that a GFD may significantly reduce depression symptoms in CD patients with classical symptoms but we are very uncertain. |
| Subgroup-asymptomatic | - | SMD 0.06 lower  (0.38 lower to 0.26 higher)  *p* = 0.71 | - | 75  (3 non-randomised studies) | ⨁◯◯◯  VERY LOW ^h,k,l^ | Upon subgroup analysis, the evidence suggests that a GFD has no significant effect on depression symptoms in asymptomatic CD patients but we are very uncertain. |
| Change in no. of CD patients positive for depression from baseline follow up: Mean one year | 495 per 1000 | 213 per 1000  (89 to 515)  *p* = 0.003 | RR 0.43  (0.18 to 1.04) | 110  (3 non-randomised studies) | ⨁◯◯◯  VERY LOW ^m,n,o^ | A GFD may significantly reduce the number of CD patients positive for depression but we are very uncertain. |
| *** The risk in the intervention group** (and its 95% confidence interval) is based on the assumed risk in the comparison group and the **relative effect** of the intervention (and its 95% CI).  **CI:** Confidence interval; **SMD:** Standardised mean difference; **RR:** Risk ratio | | | | | | |
| **GRADE Working Group grades of evidence**  **High certainty:** We are very confident that the true effect lies close to that of the estimate of the effect  **Moderate certainty:** We are moderately confident in the effect estimate: The true effect is likely to be close to the estimate of the effect, but there is a possibility that it is substantially different  **Low certainty:** Our confidence in the effect estimate is limited: The true effect may be substantially different from the estimate of the effect  **Very low certainty:** We have very little confidence in the effect estimate: The true effect is likely to be substantially different from the estimate of effect | | | | | | |

^a^ Six studies were at a high risk of detection bias, four at a high risk of confounding bias, two at a high risk of selection bias and reporting bias, and one at a high risk of bias due to deviations from interventions and commercial funding. ^b^ Moderate statistical heterogeneity. ^c^ Studies differed in population, with CD and non-CD patients, however there was no significant difference in results after subgroup analysis. ^d^ Studies used different questionnaires to assess depression, but each was self-administered and directly assessed depressive symptoms. ^e^ Large sample size and narrow CI for outcome. ^f^ The study was not blinded for participants or personnel; serious risk of detection bias. ^g^ One small study with few patients, 95% CI overlaps no effect. ^h^ All studies were at high risk of detection bias, one was also at high risk of confounding bias and bias due to deviations from intended interventions, and another was also at high risk of reporting bias. ^i^ Low-moderate statistical heterogeneity. ^j^ Large sample size but 95% CIs were estimated/imputed for one study. ^k^ Zero statistical heterogeneity. ^l^ All studies used the same questionnaire. ^m^ Two studies were at a high risk of detection and reporting bias, one at high risk of attrition bias and one at high risk of confounding bias. ^n^ High statistical heterogeneity. ^o^ All participants were newly diagnosed CD patients.

**Table S10.** Summary of findings for the short-term effects of a gluten challenge compared to placebo on depressive symptoms.

| **A Placebo (GFD) Compared to a Gluten Challenge for Depressive Symptoms (Short-Term Effects from RCTs)** | | | | | | |
| --- | --- | --- | --- | --- | --- | --- |
| **Patient or population**: Any  **Intervention**: A placebo (GFD)  **Comparison**: A gluten challenge  **Follow up:** Short-term (<1 week per intervention)  **Study type:** RCTs | | | | | | |
| **Outcomes** | **Anticipated absolute effects * (95% CI)** | | **Relative Effect (95% CI)** | **No. of Participants (Studies)** | **Certainty of the Evidence (GRADE)** | **Comments** |
|  | **Risk with a Gluten Challenge** | **Risk with a Placebo (GFD)** |  |  |  |  |
| Difference in mean depression scores between interventions | - | SMD 0.21 lower (0.58 lower to 0.15 higher)  *p* = 0.25 | - | 79  (2 RCTs) | ⨁⨁⨁◯  MODERATE ^1,a,b,c,d,e^ | A gluten challenge probably results in a small possibly insignificant effect increasing mean depression scores compared to placebo. |
| *** The risk in the intervention group** (and its 95% confidence interval) is based on the assumed risk in the comparison group and the **relative effect** of the intervention (and its 95% CI).  **CI:** Confidence interval; **SMD:** Standardised mean difference | | | | | | |
| **GRADE Working Group grades of evidence**  **High certainty:** We are very confident that the true effect lies close to that of the estimate of the effect  **Moderate certainty:** We are moderately confident in the effect estimate: The true effect is likely to be close to the estimate of the effect, but there is a possibility that it is substantially different  **Low certainty:** Our confidence in the effect estimate is limited: The true effect may be substantially different from the estimate of the effect  **Very low certainty:** We have very little confidence in the effect estimate: The true effect is likely to be substantially different from the estimate of effect | | | | | | |

^a^ One study was at high risk of reporting bias and one study was at high risk of bias due to commercial funding. ^b^ Low statistical heterogeneity. ^c^ Both studies were on non-CD participants with suspected NCGS. ^d^ Small-moderate sample size with moderate 95% CIs. ^e^ A conference abstract was found for a similar study with no significant effect of gluten on depressive outcomes.

**Table S11.** Summary of findings for the effects of compliance to a GFD on depressive symptoms.

| **Compliance Compared to Noncompliance for Depressive Symptoms** | | | | | | |
| --- | --- | --- | --- | --- | --- | --- |
| **Patient or population**: Any  **Intervention**: Compliance to GFD  **Comparison**: Noncompliance to GFD  **Follow up:** Any  **Study type:** Any | | | | | | |
| **Outcomes** | **Anticipated Absolute Effects * (95% CI)** | | **Relative Effect (95% CI)** | **Number of Participants (studies)** | **Certainty of the Evidence (GRADE)** | **Comments** |
|  | **Risk with Noncompliance** | **Risk with Compliance** |  |  |  |  |
| Difference in depression scores following a GFD—CD adults follow up: Mean one year | - | SMD 0.15 lower  (0.32 lower to 0.62 higher)  *p* = 0.54 | - | 84  (1 non-randomised study) | ⨁◯◯◯  VERY LOW ^a,b,c,d,e^ | The evidence suggests that there may be no significant effect of adherence to a GFD on depression outcomes for adult CD patients at one year, although this is very uncertain. |
| Difference in depression scores following a GFD—CD children follow up: Mean one year | - | SMD 1.40 lower  (2.38 lower to 0.42 lower)  *p* = 0.005 | - | 24  (1 non-randomised study) | ⨁◯◯◯  VERY LOW ^a,b,c,d,e^ | The evidence suggests that there may be a significant effect of adherence to a GFD on depression outcomes for paediatric CD patients at one year, although this is very uncertain. |
| Difference in depression scores long-term—CD adults follow up: Mean four years | - | SMD 0.58 lower  (1.13 lower to 0.03 lower)  *p* = 0.04 | - | 53  (1 non-randomised study) | ⨁◯◯◯  VERY LOW ^a,f^ | The evidence suggests that strict compliance to a GFD for four years may result in a larger reduction in depression compared to partial compliance. |
| *** The risk in the intervention group** (and its 95% confidence interval) is based on the assumed risk in the comparison group and the **relative effect** of the intervention (and its 95% CI).  **CI:** Confidence interval; **SMD:** Standardised mean difference | | | | | | |
| **GRADE Working Group grades of evidence**  **High certainty:** We are very confident that the true effect lies close to that of the estimate of the effect  **Moderate certainty:** We are moderately confident in the effect estimate: The true effect is likely to be close to the estimate of the effect, but there is a possibility that it is substantially different  **Low certainty:** Our confidence in the effect estimate is limited: The true effect may be substantially different from the estimate of the effect  **Very low certainty:** We have very little confidence in the effect estimate: The true effect is likely to be substantially different from the estimate of effect | | | | | | |

^a^ One study was at high risk of detection bias and reporting bias. ^b^ One study was only at high risk for detection bias. ^c^ Very high statistical heterogeneity; no overlap between 95% CIs, different directions of effect. ^d^ Use similar questionnaires to measure depression, but one study assesses CD adults and the other assesses CD children; studies assess compliance using different techniques. ^e^ One study has wide 95% CIs. ^f^ Small sample size but moderate 95% CIs.

**Table S12.** Summary of findings for GFD-treated patients’ compared to healthy controls’ depressive symptom scores.

| **Treated Patients Compared to Healthy Controls for Depressive Symptoms** | | | | | | |
| --- | --- | --- | --- | --- | --- | --- |
| **Patient or population**: Any  **Intervention**: A GFD  **Comparison**: Healthy controls | | | | | | |
| Outcomes | **Anticipated Absolute Effects^*^** (95% CI) | | **Relative Effect (95% CI)** | **Number of Participants (studies)** | **Certainty of the Evidence (GRADE)** | **Comments** |
|  | **Risk in Healthy Controls** | **Risk in Treated Patients** |  |  |  |  |
| Mean depression scores follow up: Mean one year | - | SMD 0.01  (0.18 lower to 0.20 higher)  *p* = 0.94 | - | 1268  (4 non-randomised studies) | ⨁◯◯◯  VERY LOW ^a,b,c^ | The evidence suggests there is no significant difference in depressive outcomes between patients treated with a GFD and healthy controls at one year follow-up. |
| Mean depression scores follow up: Mean four years | - | SMD 0.08 lower  (0.52 lower to 0.36 higher)  *p* = 0.72 | - | 97  (1 non-randomised study) | ⨁◯◯◯  VERY LOW ^d,e^ | The evidence suggests there is no significant difference in depressive outcomes between patients treated with a GFD and healthy controls at a four year follow-up. |
| No. of participants positive for depression follow up: Mean one year | 47 per 1000 | 253 per 1000  (109 to 585)  *p* = 0.26 | RR 5.43  (2.35 to 12.57) | 226  (2 non-randomised studies) | ⨁⨁◯◯  LOW ^f^ | The evidence suggests that there is a significantly greater risk of depression in patients treated with a GFD for one year compared to healthy controls. |
| No. of participants positive for depression follow up: Mean four years | 14 per 1000 | 111 per 1000  (12 to 1000)  *p* = 0.12 | RR 7.78  (0.85 to 71.55) | 97  (1 non-randomised study) | ⨁◯◯◯  VERY LOW ^e,g^ | The evidence suggests that there may be a greater risk of depression in patients treated with a GFD for four years compared to healthy controls but we are very uncertain about this effect. |
| *** The risk in the intervention group** (and its 95% confidence interval) is based on the assumed risk in the comparison group and the **relative effect** of the intervention (and its 95% CI).  **CI:** Confidence interval; **SMD:** Standardised mean difference; **RR:** Risk ratio | | | | | | |
| **GRADE Working Group grades of evidence**  **High certainty:** We are very confident that the true effect lies close to that of the estimate of the effect  **Moderate certainty:** We are moderately confident in the effect estimate: The true effect is likely to be close to the estimate of the effect, but there is a possibility that it is substantially different **Low certainty:** Our confidence in the effect estimate is limited: The true effect may be substantially different from the estimate of the effect  **Very low certainty:** We have very little confidence in the effect estimate: The true effect is likely to be substantially different from the estimate of effect | | | | | | |

^a^ All studies were at a high risk of detection bias, three of confounding bias, two of selection bias and reporting bias, and one for bias due to deviations from intended interventions. ^b^ Studies differed in population, with CD and non-CD patients, however there was no heterogeneity between the results of the included studies. ^c^ Studies used different questionnaires to assess depression, with one study at risk of bias due to illness. ^d^ Study at risk of detection bias and reporting bias. ^e^ One study with small sample size. ^f^ All studies at risk of detection bias and reporting bias, and one at risk of attrition bias. g. Study at risk of detection bias and reporting bias.
