# Supplementary material for: Mood Disorders and Gluten: It’s Not All in Your Mind! A Systematic Review with Meta-Analysis
Source: Nutrients. 2018 Nov 8;10(11):1708. doi: 10.3390/nu10111708 (PMC6266949; doi:10.3390/nu10111708)
Supplement: Supplementary file 1 [file nutrients-10-01708-s001.zip › nutrients-380101-supplementary proof/Supplementary File 6 Edited.docx]

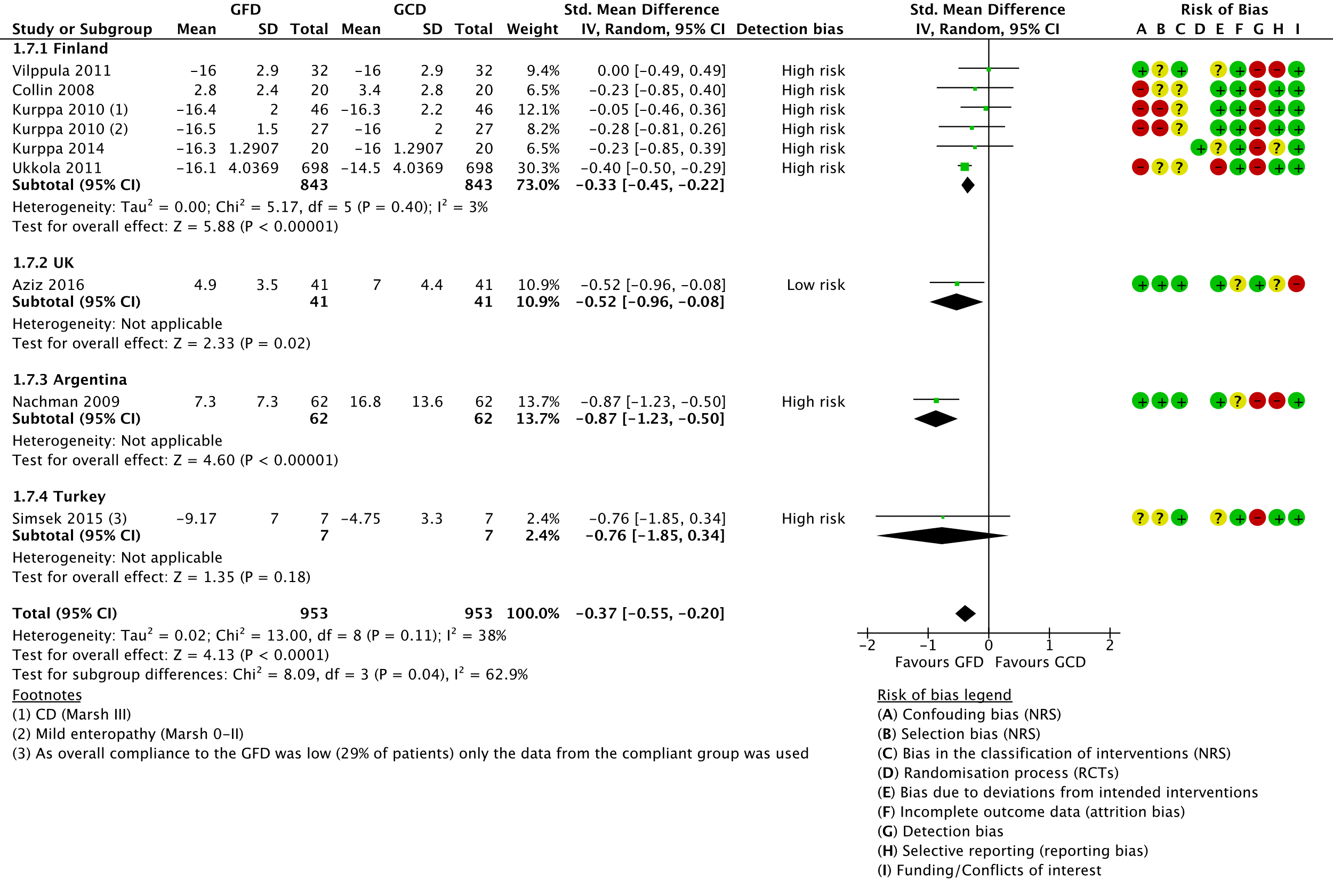

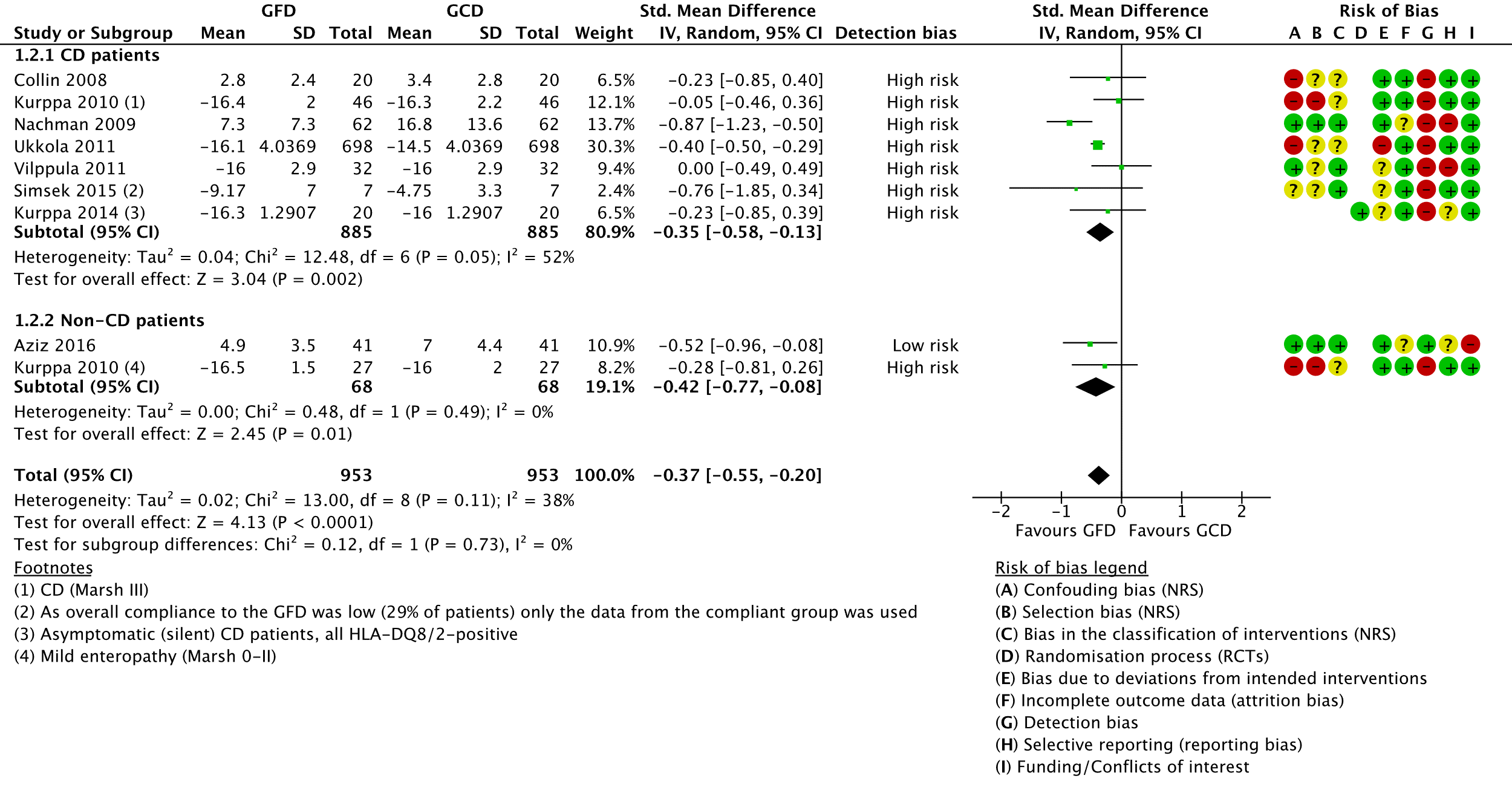

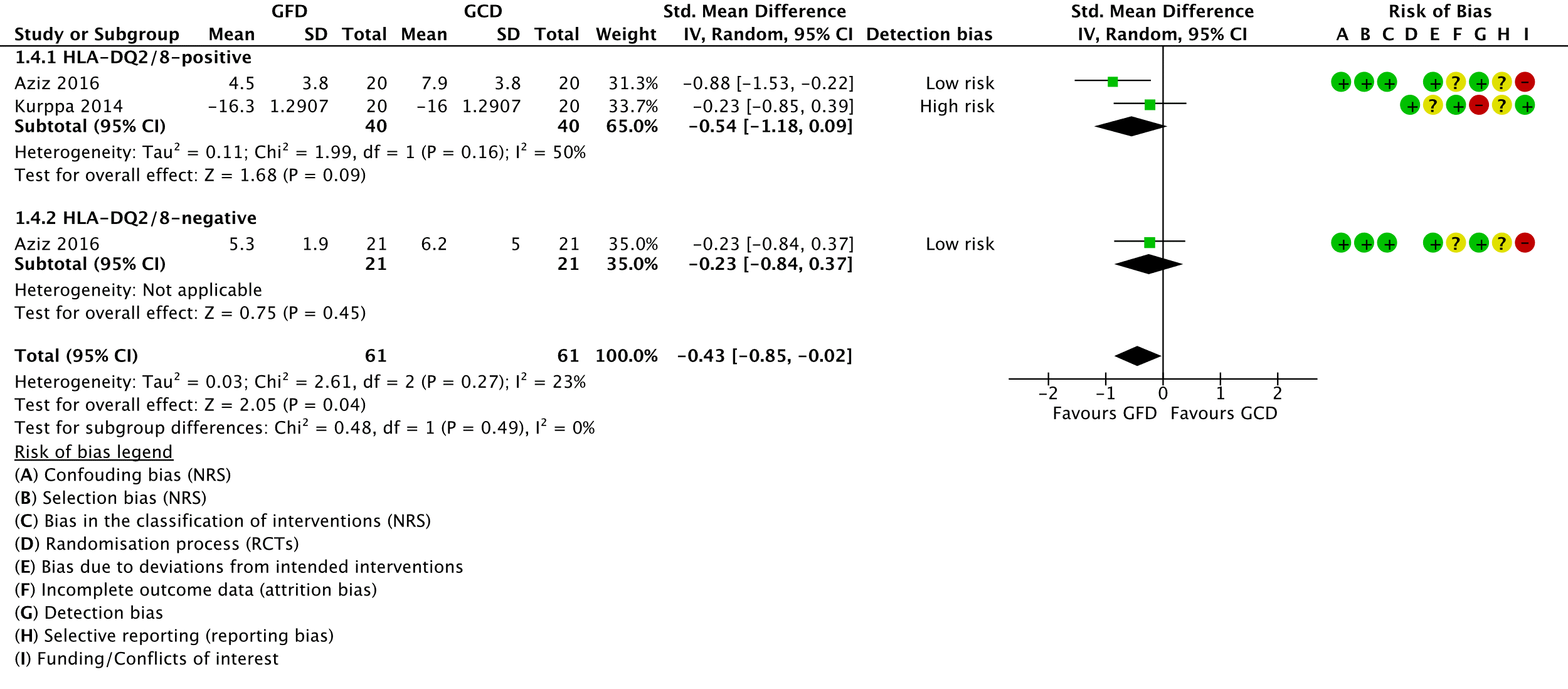


Figure S1. Forest plots demonstrating the difference in mean depression scores between following a GFD (gluten-free diet) and a GCD (gluten-containing diet) for the following subgroup analyses: (A) Population country of origin; (B) coeliac disease (CD) diagnosis—CD and non-CD patients; and (C) HLA-DQ2/8 genotype—positive and negative.

**B**

**A**

**A**

**B**

**C**

**C**


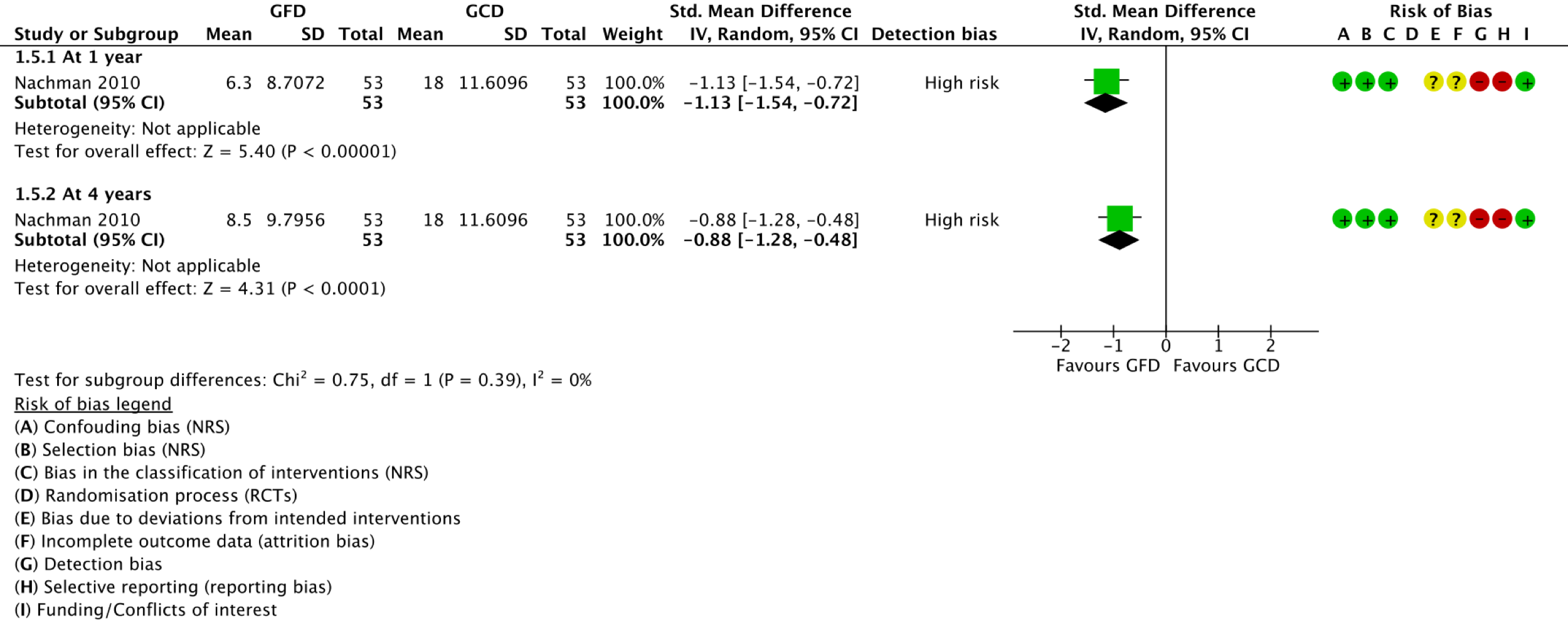


Figure S2. Forest plot demonstrating the relative difference in change of mean depression scores between baseline + year four and baseline + year four for participants following a GFD for four years.


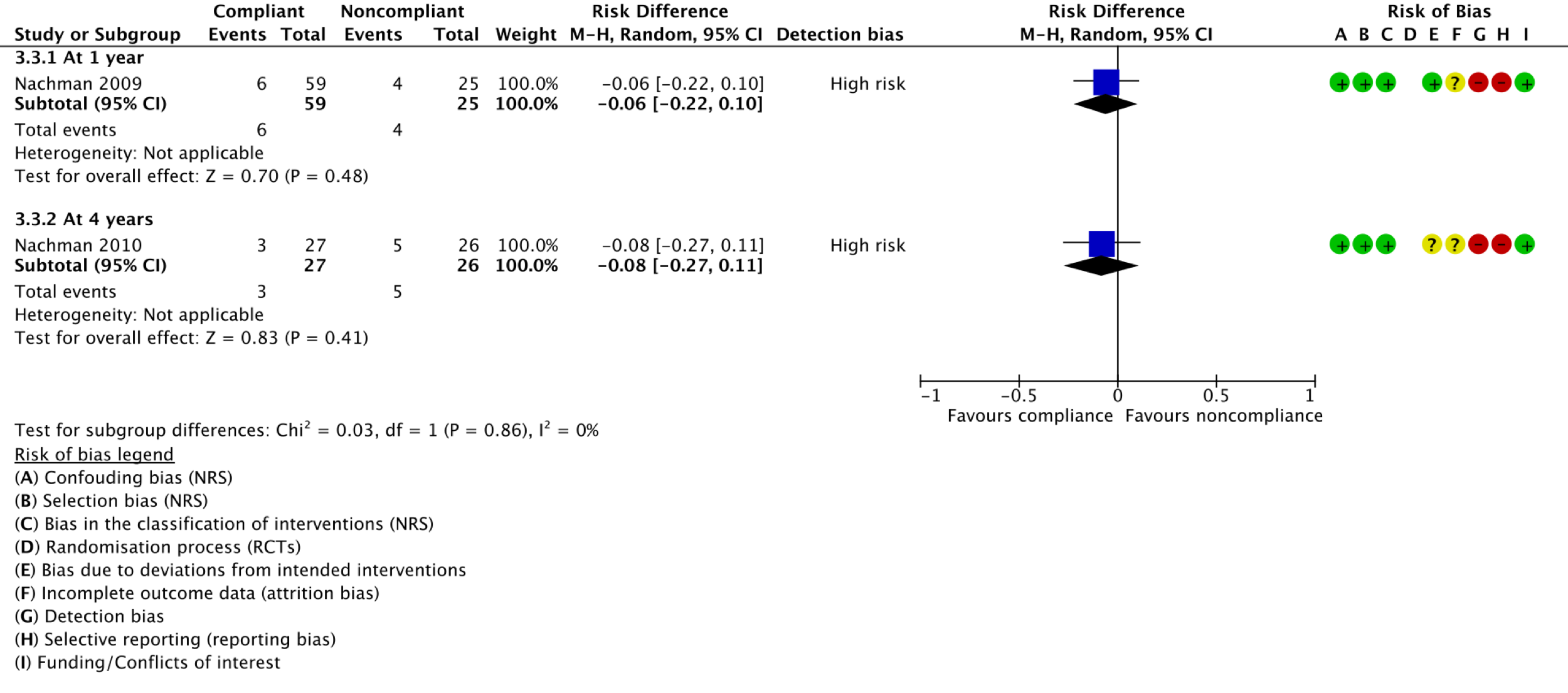


Figure S3. Forest plots comparing the numbers of CD adult patients positive for depression at one year and four years.
