# Supplementary material for: Mood Disorders and Gluten: It’s Not All in Your Mind! A Systematic Review with Meta-Analysis
Source: Nutrients. 2018 Nov 8;10(11):1708. doi: 10.3390/nu10111708 (PMC6266949; doi:10.3390/nu10111708)
Supplement: Supplementary file 1 [file nutrients-10-01708-s001.zip › nutrients-380101-supplementary proof/Supplementary File 7 Checked.docx]

**Table S13.** Sensitivity analysis comparing random-effects (RE) and fixed-effects (FE) models

| Comparison | SMD/RD | | 95% CI | | *P*-value | |
| --- | --- | --- | --- | --- | --- | --- |
|  | **RE** | **FE** | **RE** | **FE** | **RE** | **FE** |
| 3.4.1.1. | -0.37 | -0.39 | -0.55 to -0.20 | -0.48 to -0.30 | <0.0001 | <0.00001 |
| 3.4.1.2. | -0.31 | -0.34 | -0.52 to -0.10 | -0.46 to -0.23 | 0.003 | <0.00001 |
| 3.4.2. | 0.21 | 0.19 | -0.58 to 0.15 | -0.12 to 0.51 | 0.25 | 0.22 |
| 3.4.4.1. at 1 year | 0.01 | 0.01 | -0.18 to 0.20 | -0.18 to 0.20 | 0.94 | 0.94 |
| 3.4.4.1. at 4 years | -0.08 | -0.08 | -0.52 to 0.36 | -0.52 to 0.36 | 0.72 | 0.72 |
| 3.4.4.2. at 1 year | 0.21 | 0.19 | -0.16 to 0.58 | 0.10 to 0.27 | 0.26 | <0.0001 |
| 3.4.4.2. at 4 years | 0.10 | 0.10 | -0.02 to 0.22 | -0.02 to 0.22 | 0.12 | 0.12 |

**Table S14.** Sensitivity analysis comparing risk difference (RD), risk ratio (RR) and odds ratio (OR) for dichotomous outcomes

| Comparison | RD | RR | OR | 95% CI | | | *P*-value | | |
| --- | --- | --- | --- | --- | --- | --- | --- | --- | --- |
|  |  |  |  | **RD** | **RR** | **OR** | **RD** | **RR** | **OR** |
| 3.4.1.2. | -0.31 | 0.43 | 0.22 | -0.52 to -0.10 | 0.18 to 1.04 | 0.06 to 0.86 | 0.003 | 0.06 | 0.008 |
| 3.4.4.2. at 1 year | 0.21 | 5.43 | 8.34 | -0.16 to 0.58 | 2.35 to 12.57 | 3.05 to 22.76 | 0.26 | <0.0001 | <0.0001 |
| 3.4.4.2. at 4 years | 0.10 | 7.78 | 8.63 | -0.02 to 0.22 | 0.85 to 71.55 | 0.86 to 86.92 | 0.12 | 0.07 | 0.07 |


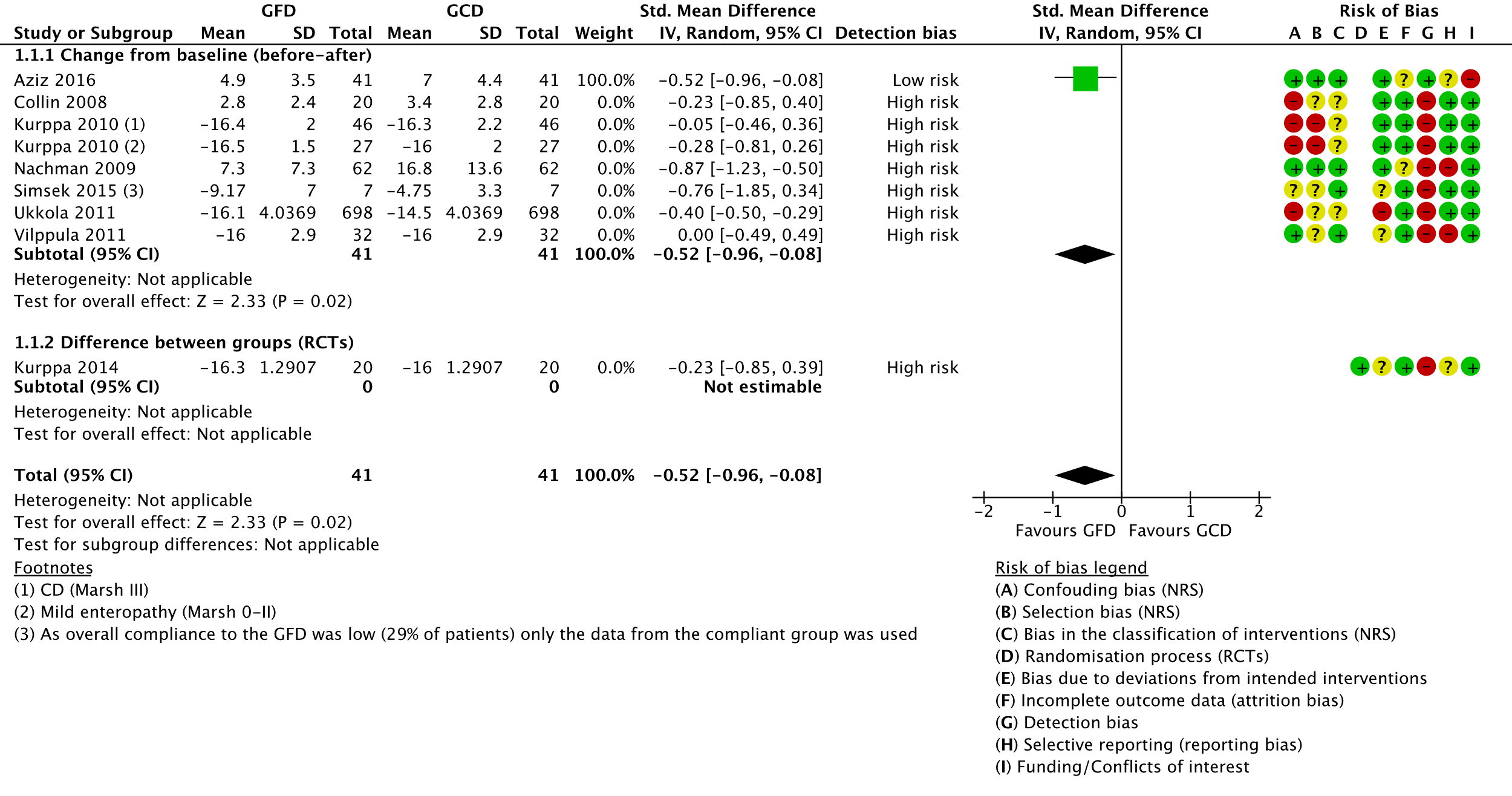

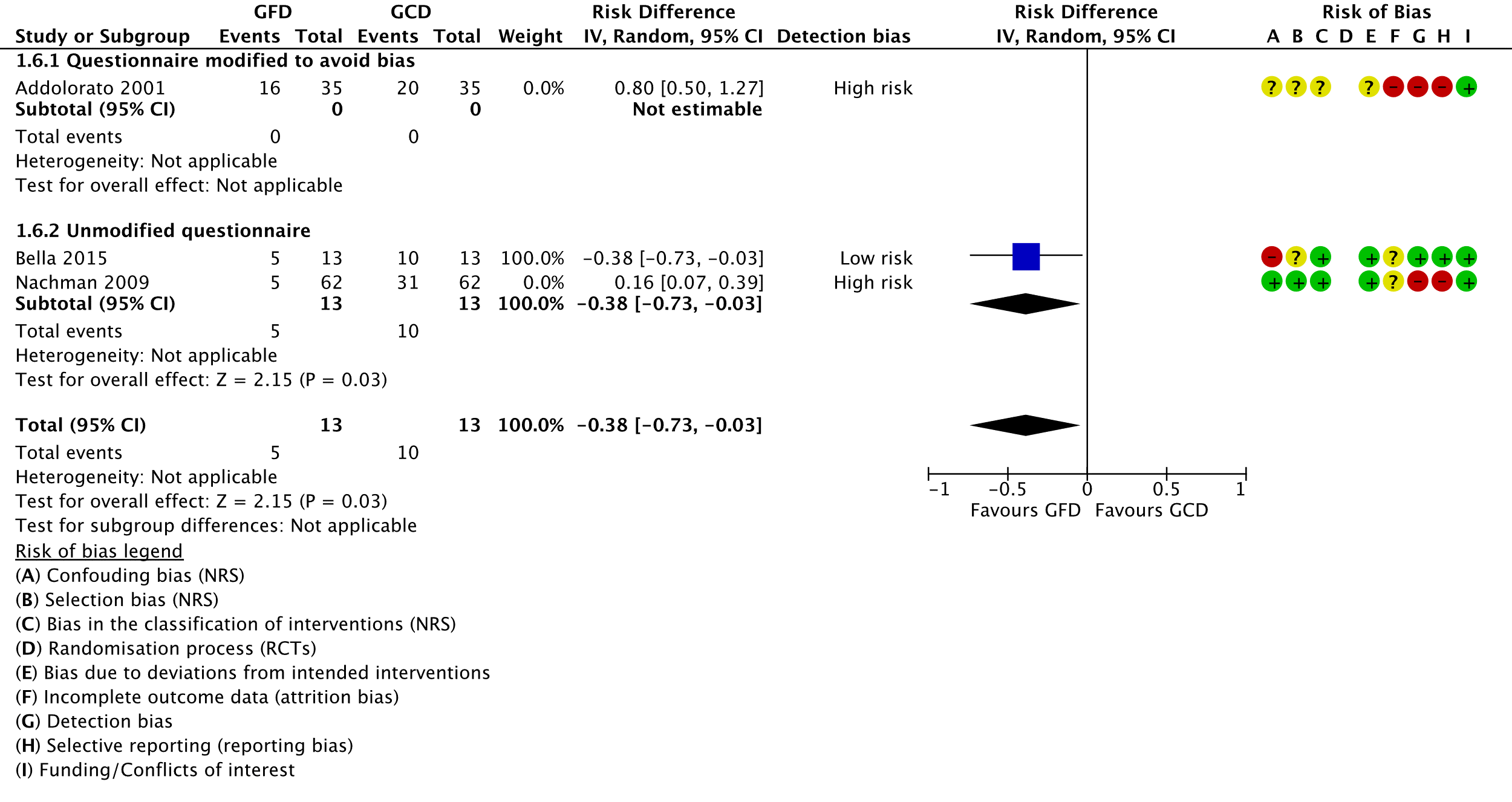


**Figure S4.** Sensitivity analysis removing studies at unclear or high risk of detection bias for comparisons (A) 3.4.1.1. and (B) 3.4.1.2.

**A**

**B**

**Figure S5.** Sensitivity analysis removing data from Nachman et al.


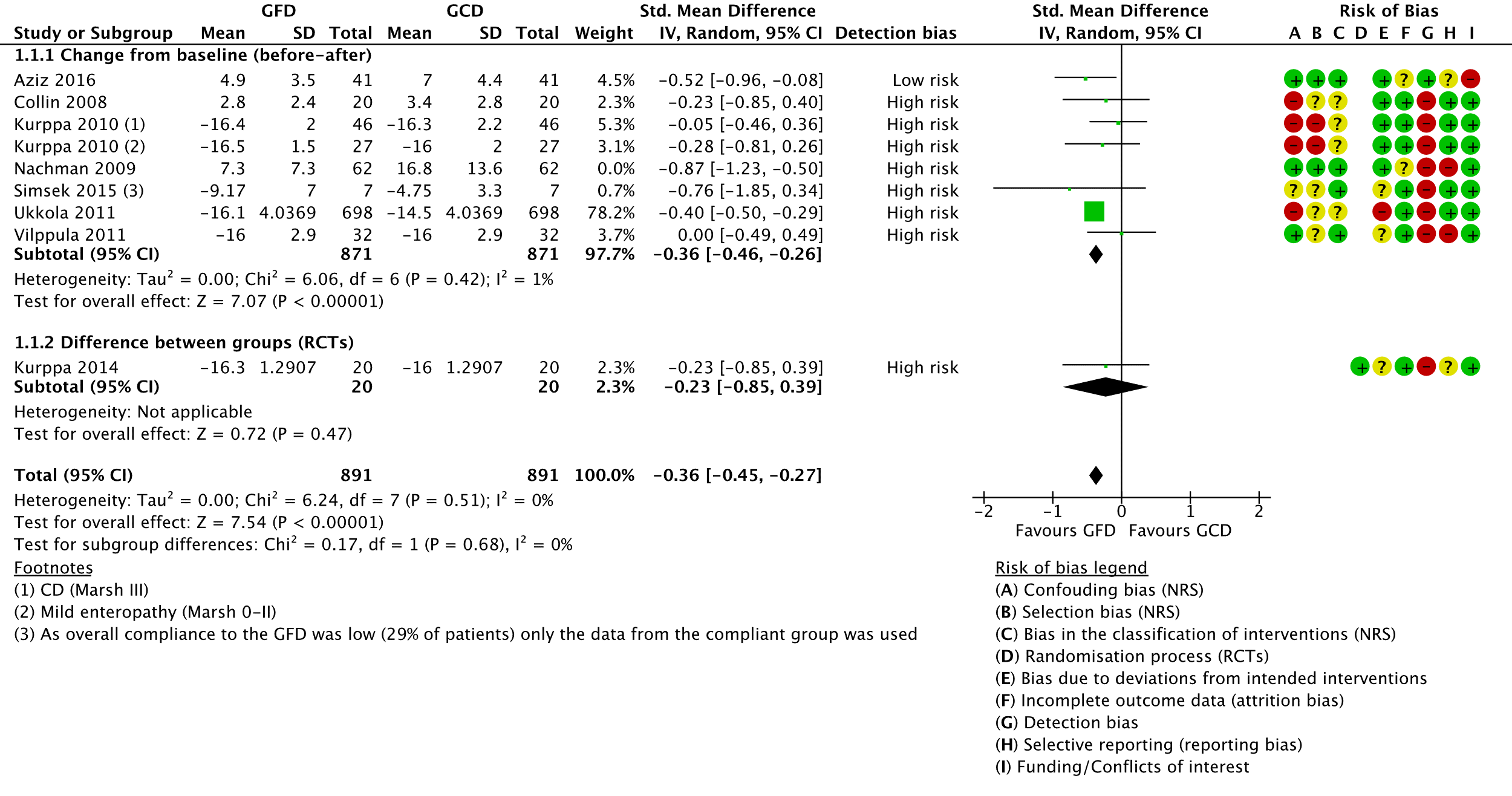

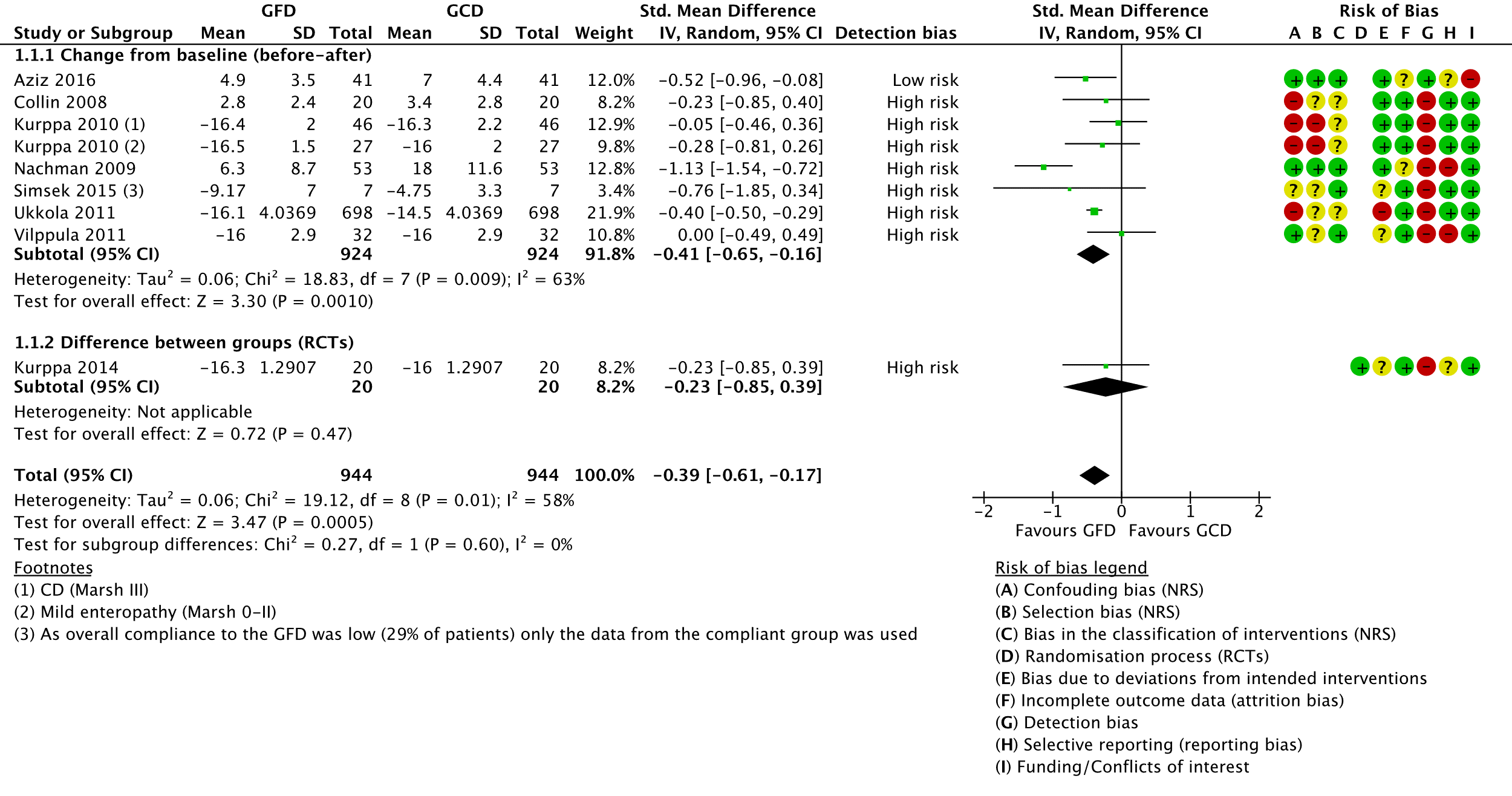

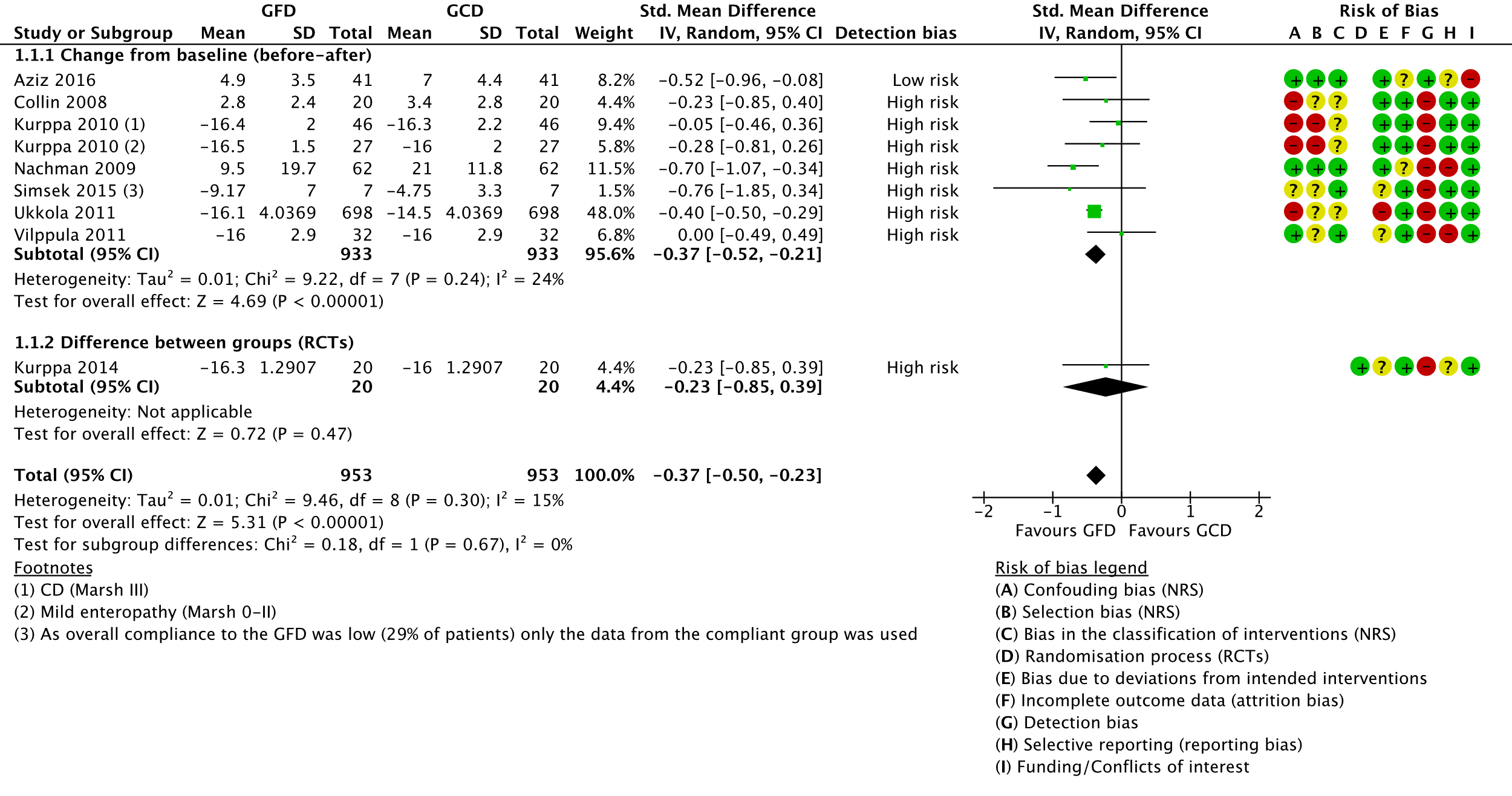


**Figure S6.** Sensitivity analysis swapping Nachman et al. (2009) outcome data for classical CD patients from the modified BDI to that for (A) 62 classical CD patients from the unmodified BDI (Nachman et al., 2009), and (B) 53 CD patients from the unmodified BDI (Nachman et al., 2010)

**A**

**B**
